# Training in normobaric hypoxia induces hematological changes that affect iron metabolism and immunity

**DOI:** 10.1038/s41598-025-01542-w

**Published:** 2025-05-22

**Authors:** Svenja Nolte, Deeksha Malhan, Anna Klemmer, Tom Kastner, Nico Walter, Daniel Fleckenstein, Johannes Keck, Simon Klügel, Celina Maier, Kristina Gebhardt, Tobias Stauber, Angela Relógio, Karsten Krüger, Karsten Hollander

**Affiliations:** 1 Department of Exercise Physiology and Sports Therapy, Institute of Sport Science, Justus-Liebig-University Giessen, Giessen, Germany; 2 Institute of Interdisciplinary Exercise Science and Sports Medicine, Medical School Hamburg, Hamburg, Germany; 3 Institute for Systems Medicine, Medical School Hamburg, Hamburg, Germany; 4 Institute for Molecular Medicine, Medical School Hamburg, Hamburg, Germany; 5 Corporate Member of Freie Universität Berlin and Humboldt-Universität zu Berlin, Department of Sports Medicine, Charité - Universitätsmedizin Berlin, Berlin, Germany; 6 Institute for Applied Training Science, Leipzig, Germany

**Keywords:** Neutrophils, Athletes, Hypoxia, Transferrin, Lactoferrin, Metabolism, Cytokines

## Abstract

Altitude training is a method among endurance athletes to enhance performance via hypoxia-induced adaptations. However, individual responses vary significantly, with some athletes even showing performance decrements. Iron metabolism and immune function may influence these adaptations, as hypoxia-induced erythropoiesis increases systemic iron demand, potentially affecting immune cells reliant on iron. This study investigated the interplay between hematological, iron, and immunological variables under controlled normobaric hypoxia. 15 highly trained athletes participated in a 21-day live-high-train-low training camp in a normobaric altitude house. Blood samples were collected pre- and post-camp and at four intermediate time points to measure hematological variables, iron metabolism variables, and immunological variables. Pre- and post-performance was assessed via VO_2_max tests. Statistical analyses included paired t-tests, Wilcoxon rank-sum test, Spearman correlations, and Granger causality analysis to explore systemic temporal interactions. VO_2_max increased significantly (*p* < 0.05) with large interindividual variability (2.4 ± 3.5 ml/min/kg). Hemoglobin concentration, erythrocytes, and the soluble transferrin receptor (sTfR) showed significant increases over time (*p* < 0.05), while ferritin peaked early and declined post-camp. Myeloperoxidase and lactoferrin exhibited dynamic correlations with iron variables (*p* < 0.05), reflecting competition between erythropoiesis and immune function for iron. The structure of the Granger causality network places transferrin in a central role, highlighting iron metabolism as one key regulator of these adaptations. Normobaric hypoxia training induces systemic physiological changes involving hematological, iron, and immune systems. Controlled hypoxic conditions enable detailed exploration of these interactions, providing insights into optimizing altitude training strategies for endurance performance enhancement.

## Introduction

Altitude training camps are a widely utilized method among elite athletes aiming to enhance endurance performance. These camps expose athletes to hypoxic conditions, which provides an additional stimulus to adapt the oxygen transport capacity. Accordingly, numerous adaptations of the hematological system have been shown to have a positive effect on performance in endurance sports^1^.

However, the literature highlights a very individual response to hypoxia-induced altitude exposure. While some athletes experience substantial performance gains, others show minimal improvements or even suffer performance decrements^2–4^, maybe due to an overreaching of their physiological capacity to adapt to these demanding conditions.

One of the primary mechanisms proposed to underpin performance enhancement from altitude exposure is the increase in total hemoglobin mass (Hb_mass_), which augments oxygen transport capacity^5^. However, the causal factors for driving Hb_mass_ elevation remain insufficiently understood, and the broader physiological consequences of these changes on other systems, such as iron metabolism and immune function, are inadequately explored. Investigating the system-physiological interactions may help better understand the variability in individual responses to altitude training^6^.

Iron plays a pivotal role in the physiological adaptation to hypoxia, primarily by supporting erythropoiesis. Hypoxia-induced erythropoiesis significantly increases iron demand, which can deplete systemic iron stores^7^. This in turn could have consequences for other organs or tissues that are also dependent on iron and also have a physiological significance for performance. Studies that have used omics analyses have expanded the understanding of iron metabolism and its systematic consequences, such as the immune system^8^. It is well known that neutrophils rely on iron to maintain their antimicrobial functions. In particular, myeloperoxidase (MPO) production is highly iron-dependent and can be impaired by iron deficiency^9^. The heightened competition for iron between erythropoiesis and immune functions under physiological stress and hypoxic conditions represents a physiological challenge that can influence performance development as well as overall health.

In training camps in hypobaric hypoxia, where athletes usually train, it is very difficult to create controlled conditions. Camps in normobaric hypoxia provide more controlled conditions because hypoxia effects are isolated from confounding environmental variables such as UV exposure and changing weather conditions. In altitude houses, athletes train and reside under precisely controlled hypoxic conditions, enabling standardized investigations of the complex physiological networks of altitude adaptations^10^.

The aim of our study was to investigate the interplay between hematological changes, iron metabolism, and the immune system under the controlled conditions of normobaric hypoxia in national level athletes. We hypothesized that training at altitude would lead to a competitive situation for iron between erythropoiesis and neutrophil function, which may partly explain the training response’s variability. By elucidating these relationships, we aim to contribute to a more comprehensive understanding of the physiological adaptations to hypoxia and implement individualized altitude training strategies.

## Methods

### Study design

**Fig. 1:**
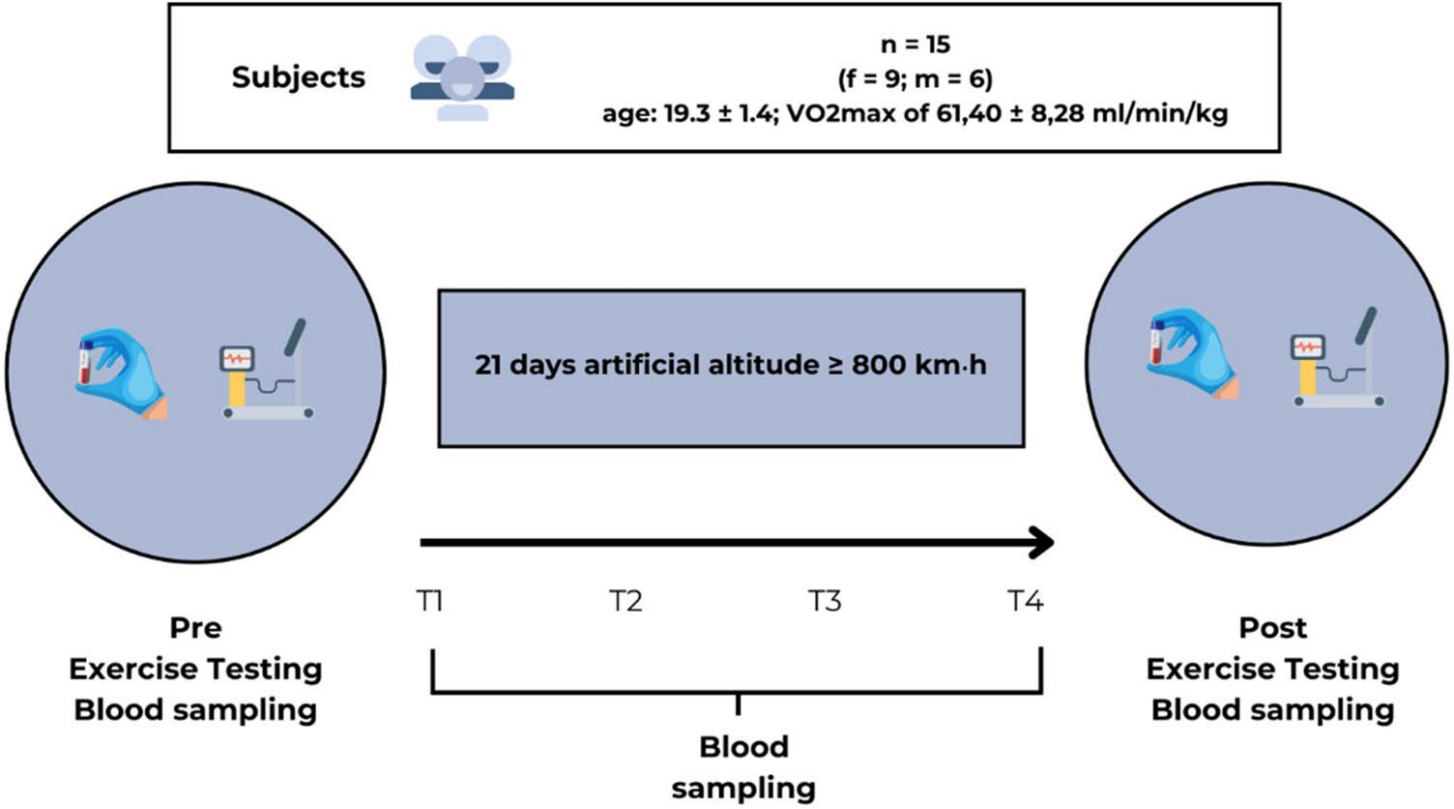
Study design of the normobaric hypoxia training camp. The procedure applies to every group of athletes.

The study included three 21-days-training camps in normobaric hypoxia. In every camp, four to six athletes were monitored. All athletes (except one) supplemented iron in the lead-up to camp, which followed an individual approach that was monitored by medical staff and coaches. The altitude camp employed a semi-structured live-high-train-low approach with a minimum of 800 km·h of normobaric hypoxia (Höhenbalance GmbH, Going, Austria). Athletes spent a minimum of 14 h daily in normobaric hypoxia with training sessions either within the normoxic environment or outside the altitude facility. Six blood samples were collected, one a minimum of 7 days before (pre) and one 10–14 days after (post) the altitude training camp, and at four subsequent time points at altitude: 24 h (T1), 7 days (T2), 14 days (T3), and day 21 (T4) (Fig. 1).

### Subjects

National team athletes were recruited from the German Athletics Federation (Deutscher Leichtathletik Verband – DLV). Fifteen highly trained endurance athletes (n = 15 [female (f) = 9; male (m) = 6]; age: 19.3 ± 1.4 years [f = 19.4 ± 1.7 years; m = 19.2 ± 1.0 years]; VO_2_max of 61.40 ± 8.28 ml/min/kg [f = 56.95 ± 6.16 ml/min/kg; m = 68.08 ± 6.48 ml/min/kg]) from long- and middle-distance running disciplines and race walking were included in the study. Exclusion criteria were contraindications for exposure to altitude (anaemia, acute upper respiratory tract infections, chronic lung diseases (asthma only with adequate medication), cardiac insufficiency, myocarditis (in the last 6 months), epilepsy, cardiac arrhythmia). Athletes were recruited in close coordination with the coaches and were selected for the junior or perspective squad of the 2023 season. Prior to participation, all individuals were informed about the potential risks and benefits of the study and provided written informed consent, as per institutional guidelines.

The study was approved by the local ethical committees (MSH-2022/211) and was conducted following the Declaration of Helsinki for human research.

### Exercise testing

At least 1 week before and after the training camp, performance diagnostics were conducted to determine the maximal oxygen consumption (VO_2_max), the velocity where VO_2_max is reached (VO_2_max) and the velocity at volitional exhaustion (ramp test; 1 min stage duration; 0.15 m/s incline; 0% treadmill incline; individual starting speed according to performance level; test duration 6–10 min). The respiratory gases were recorded using the breath-by-breath method (MetaLyzer 3B-R2, CORTEX Biophysik GmbH, Leipzig, Germany).

### Training load

The athletes completed a 3 week training block in normobaric hypoxia. Due to the high-performance sports setting, individual training and season planning, no intervention was applied in the training process. Training organisation remained the responsibility of the coach and the athletes. Guidance regarding training zones (based on prior performance diagnostics) was provided to the athletes for training monitoring. The training sessions were individually regulated using heart rate and lactate measurements. All athletes were at the camp during an early phase of the season (preparatory phase), with a primary focus on low-intensity training.

For every training session, the session-RPE (rating of perceived exertion) was calculated for the purpose of recording the individual training load of the athletes^11,12^. This method considers both the intensity and the duration of a training session, on a scale of RPE multiplied by the training duration. The calculated score was recorded for each athlete individually to determine the training load for each day and to calculate an average value over the duration of the stay in normobaric hypoxia. Furthermore, the ‘session RPE’ was calculated for the 4-week period preceding the altitude training to ascertain whether and to what extent training loads were reduced or increased during the altitude training. A ratio analogous to the acute:chronic workload ratio (ACWR) was calculated to correlate the 4-week training load prior to the training camp with the training load at altitude. The aforementioned rate of increase is incorporated into the analysis based on a factor, which is hereinafter referred to in a simplified manner as the “Training Load Ratio Altitude” (TLRA).

### Blood sampling

All venous blood samples were taken between 8 and 10 a.m. In total, 17.5 ml of blood was collected. The EDTA blood tubes were processed immediately at the camp and centrifuged at 2500 g for 10 min at room temperature. Plasma was separated into aliquots and stored in Eppendorf containers, which were then stored on dry ice for transport and frozen at − 80 °C.

### Hematological variables

To determine the number of erythrocytes, lymphocytes, thrombocytes, neutrophils, reticulocytes and hemoglobin concentration the small EDTA vacutainer was analyzed by routine clinical laboratory methods by SYNLAB Medical Care Center (Bad Nauheim, Germany). Erythropoietin (EPO) levels in 50-µl blood plasma samples were measured in duplicates by enzyme-linked immunosorbent assay (ELISA) using the Human EPO ELISA Kit (Thermo Fisher Scientific, Waltham, MA, USA) according to the manufacturer’s instructions. Signal was detected with an Infinite M Plex plate reader (TECAN, Männedorf, Switzerland) at 450 nm excitation.

### Iron metabolism variables

Ferritin, transferrin, transferrin saturation, hepcidin, soluble transferrin receptor (sTfr) and Ferritin index (sTfR/log ferritin) were analyzed using the serum gel vacutainer and were also analyzed by routine clinical laboratory methods by SYNLAB Medical Care Center (Bad Nauheim, Germany).

### Plasma immune variables

CD163, TNF-alpha, VEGF, IL-6, IL-10, BDNF, IL-1 beta, IL-1ra, IL-8, S100A8, myeloperoxidase (MPO) and lactoferrin levels in 50-µl blood plasma samples were measured in duplicates by enzyme-linked immunosorbent assay (ELISA) using the magnetic Luminex assay (Bio-Techne Ltd, Abingdon, Oxon, UK).

### Statistics

Before carrying out further statistical analysis the Shapiro–Wilk test was performed to test for normal distribution of all parameters. If the assumption of normality was violated (*p* < 0.05), a non-parametric test was performed. If normal distribution was present (*p* > 0.05), a parametric test was conducted.

A paired t-test was conducted to assess the change in VO_2_max before and after (ΔVO_2_max) the intervention and analyzed the effect size by calculating Cohen’s d. This test was chosen to account for the fact that the same subjects were measured at both time points, allowing for the comparison of dependent samples.

To analyze the association between ΔVO_2_max and TLRA a Spearman correlation was carried out. The Wilcoxon rank-sum test was performed to determine whether the parameters showed significant differences at different time points. Additionally, a Spearman correlation was carried out to analyze associations between hematological, iron metabolism, and immune variables, as this method does not require the assumption of normality.

A Granger network analysis was conducted to explore temporal interconnections between the individual parameters, allowing for a deeper understanding of the causal relationships among the variables. The analysis was carried out in several steps. First, a representative time series for each sample was calculated using robust scaling within each parameter across all subjects to minimize potential influence of outliers. Next, stationarity of the time series was assessed using the Kwiatkowski-Phillips-Schmidt-Shin (KPSS) test. This test was critical for ensuring that the data met the necessary assumptions for Granger causality analysis, as non-stationary data could lead to misleading conclusions. This step aimed to determine whether incorporating part values of one parameter (time lag = 1) enhances the prediction of future values of another parameter, and vice versa. This approach allowed for the exploration of temporal relationships in both directions. the prediction of future values of one variable is increased by incorporating past values of another (time lag = 1), and vice versa, allowing for the exploration of temporal relationships in both directions. Finally, significant Granger causal connections were identified by determining whether incorporating the past values of one parameter improved the prediction of future values compared to using only the parameter itself. Only those relationships that showed statistically significant causal links after correction for multiple testing were retained for further analysis, providing a refined network of temporal associations among the parameters^13^.

## Results

### Training characteristics, training load ratio and VO_2_max

The average weekly training time was 14.52 ± 3.36 h. The number of training sessions per week was recorded at 12.98 ± 2.14 (including physiotherapy and recovery sessions), and strength training was reported at 1.32 ± 0.45 h. Specific training time (running and/or race walking) amounted to 6.01 ± 1.48 h per week, with a contribution of intensive training kilometres by 13.2 ± 12.0%. Additionally, athletes spent an average of 142 ± 88 min per day outside the altitude house. For TLRA, an average value, across all athletes, of 1.52 ± 0.41 was observed. The lowest ratio recorded was 0.98, and the highest was 2.35. This indicates that, during their time in normobaric hypoxia, the athletes increased their training load by an average of 52% compared to the 4 weeks before. However, individual variability was relatively high: one athlete maintained nearly the same load (− 2%), while another athlete increased their load by 135%. The effect on the development of performance at altitude was measured by the change in VO_2_max (ΔVO_2_max) between pre and post. The results showed that VO_2_max increased by 2.1 ± 1.9 ml/min/kg (*p* < 0.05, d = 0.69) after 21 training days at altitude. However, subjects demonstrated high inter-individual differences between a 1.7 ml/min/kg decrease and an 4.6 ml/min/kg increase. No associations were found between the TLRA and ΔVO_2_max.

### Hematological variables

**Fig. 2:**
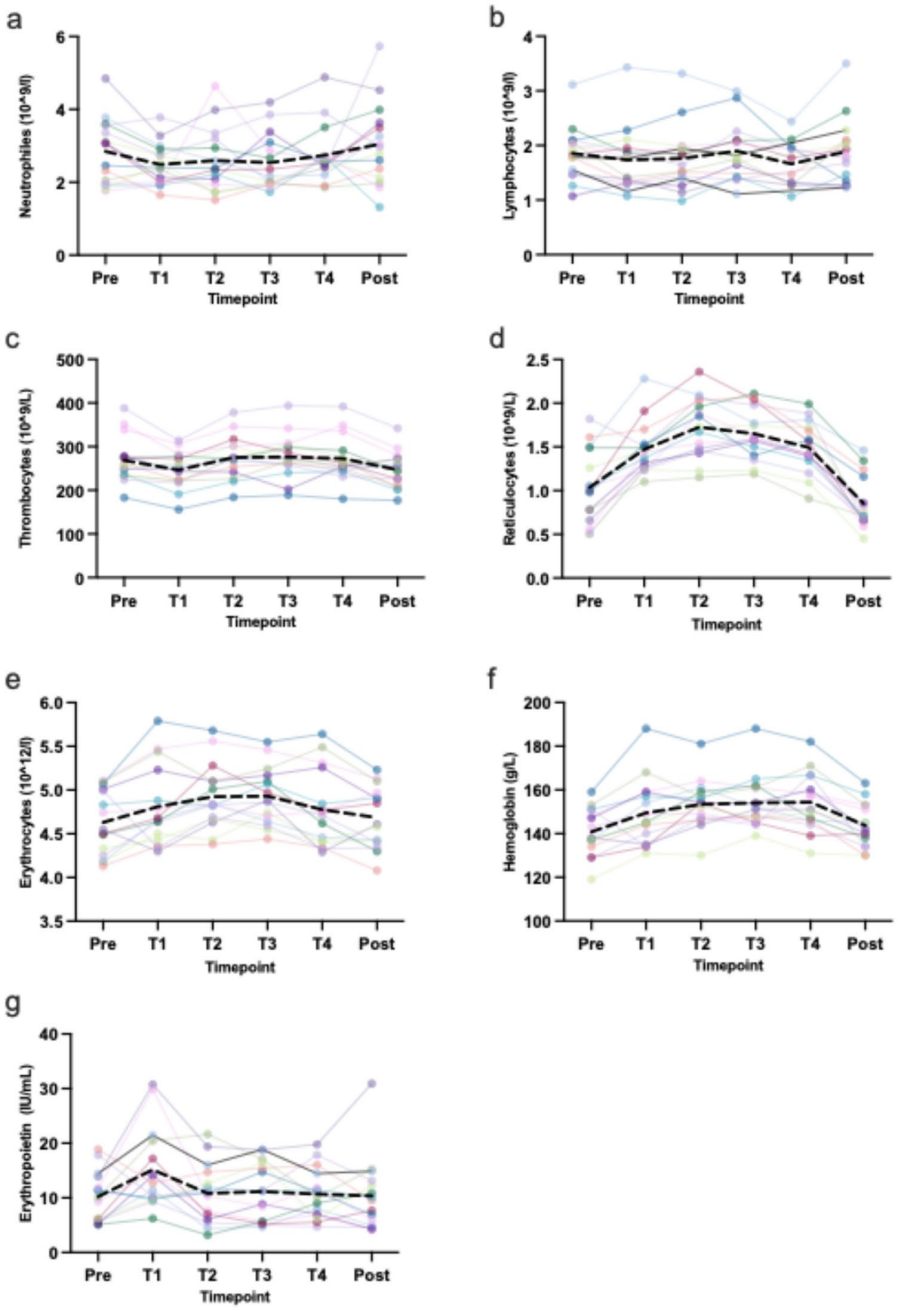
Changes of hematological variables over time. Shown are individual progressions (colored lines) and the mean progression (black dotted line).

Number of neutrophils showed a marginal decrease at altitude (pre 2.85 ± 0.90, T1 2.49 ± 0.59 10^9^/l), followed by an increase at T4 (3.05 ± 1.13 10^9^/l) (Fig. 2a). In contrast, the number of lymphocytes demonstrated no statistically significant alterations over time (Fig. 2b). Significant changes over time were found for thrombocytes (*p* < 0.05) (Fig. 2c) and reticulocytes (*p* < 0.05) (Fig. 2d), showing an increase at altitude. Similarly, the number of erythrocytes increased (pre 4.63 ± 0.37, T2 4.92 ± 0.37, post 4.68 ± 0.35 10^12^/l) without a significant time effect (Fig. 2e). Hemoglobin concentration presented a significant time effect (*p* < 0.05) (Fig. 2f) demonstrating an increase at altitude. EPO increased only within the first 24 h (pre 10.22 ± 4.59, T1 (15.16 ± 7.44) and dropped near baseline over time (T4 10.67 ± 4.62) (Fig. 2g).

### Iron metabolism variables

**Fig. 3:**
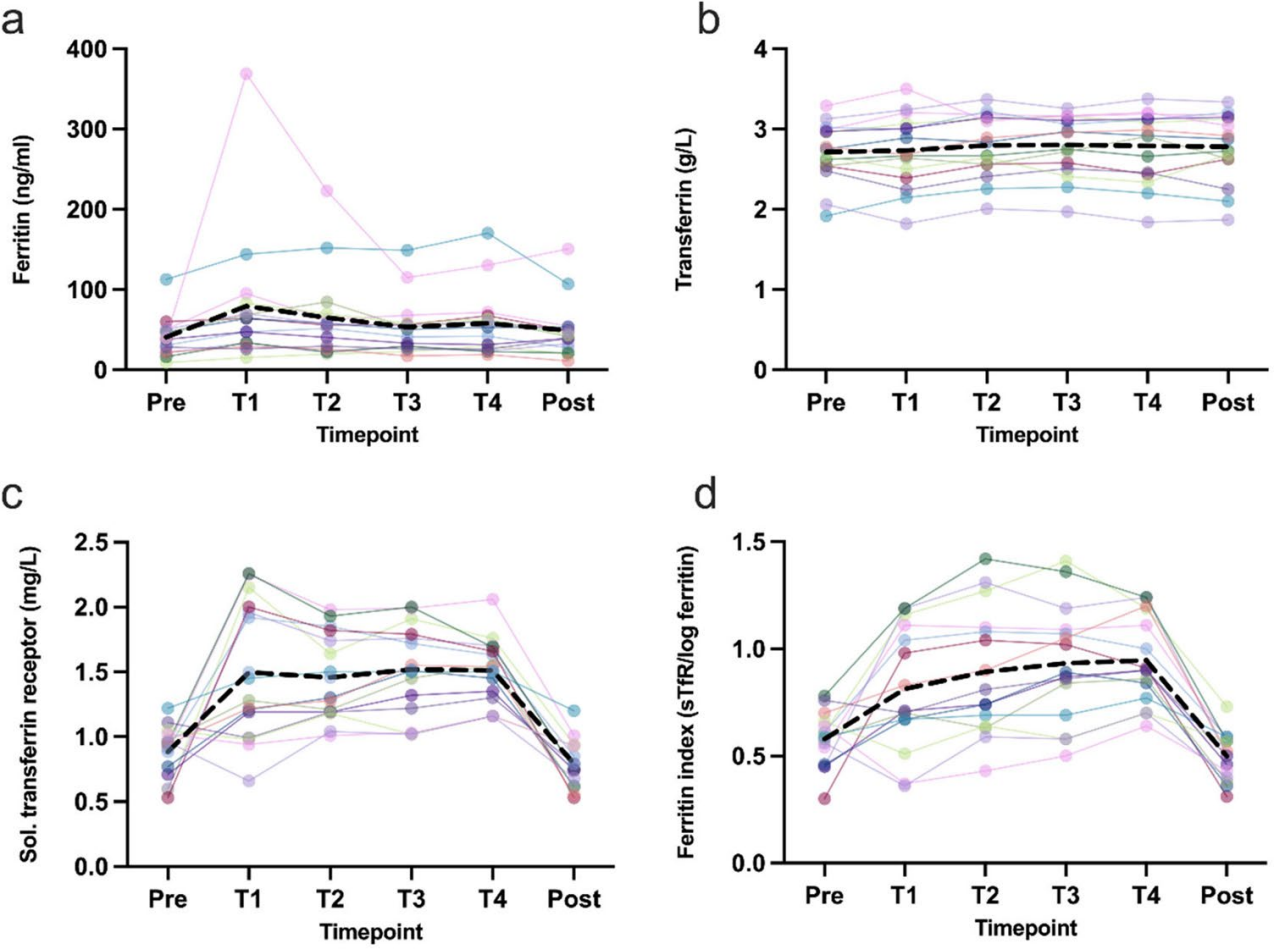
Changes of iron metabolism variables over time. Shown are individual progressions (colored lines) and the mean progression (black dotted line).

Ferritin levels increased from pre-measurement (40.92 ± 24.49 ng/ml) to T1 (79.50 ± 86.43 ng/ml), followed by a moderate decrease until post (49.22 ± 35.74 ng/ml) (Fig. 3a). While mean serum transferrin levels remained constant over time (Fig. 3b) significant changes were found for concentrations of the soluble transferrin receptor (*p* < 0.05) and the ferritin index (*p* < 0.05) over time, (Fig. 3c,d).

### Immunological variables

**Fig. 4:**
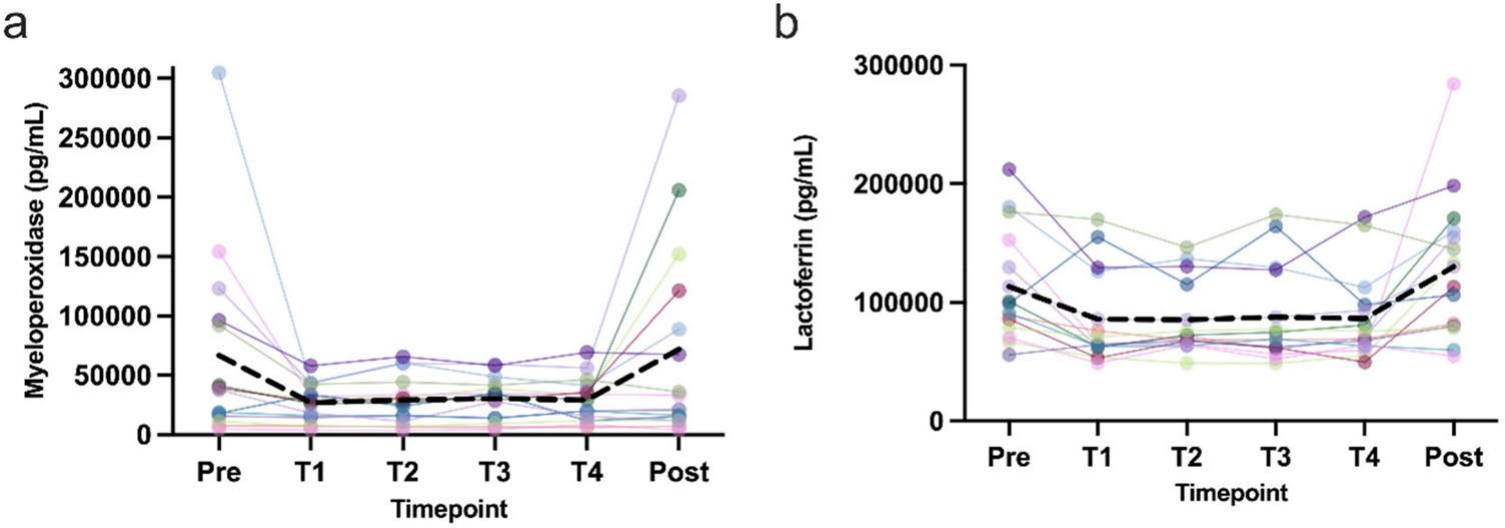
Changes of products of neutrophil activity over time. Shown are individual progressions (colored lines) and the mean progression (black dotted line).

Both parameters of neutrophil activity, MPO and lactoferrin, showed a significant time effect (*p* < 0.05) (Fig. 4a,b). No significant changes were observed for plasma levels of CD163, TNF-alpha, VEGF, IL-6, IL-10, BDNF, IL-1 beta, IL-1ra, IL-8, and S100A8 (data not shown).

### Associations between hematological variables, factors of iron metabolism and immunological parameters

**Fig. 5:**
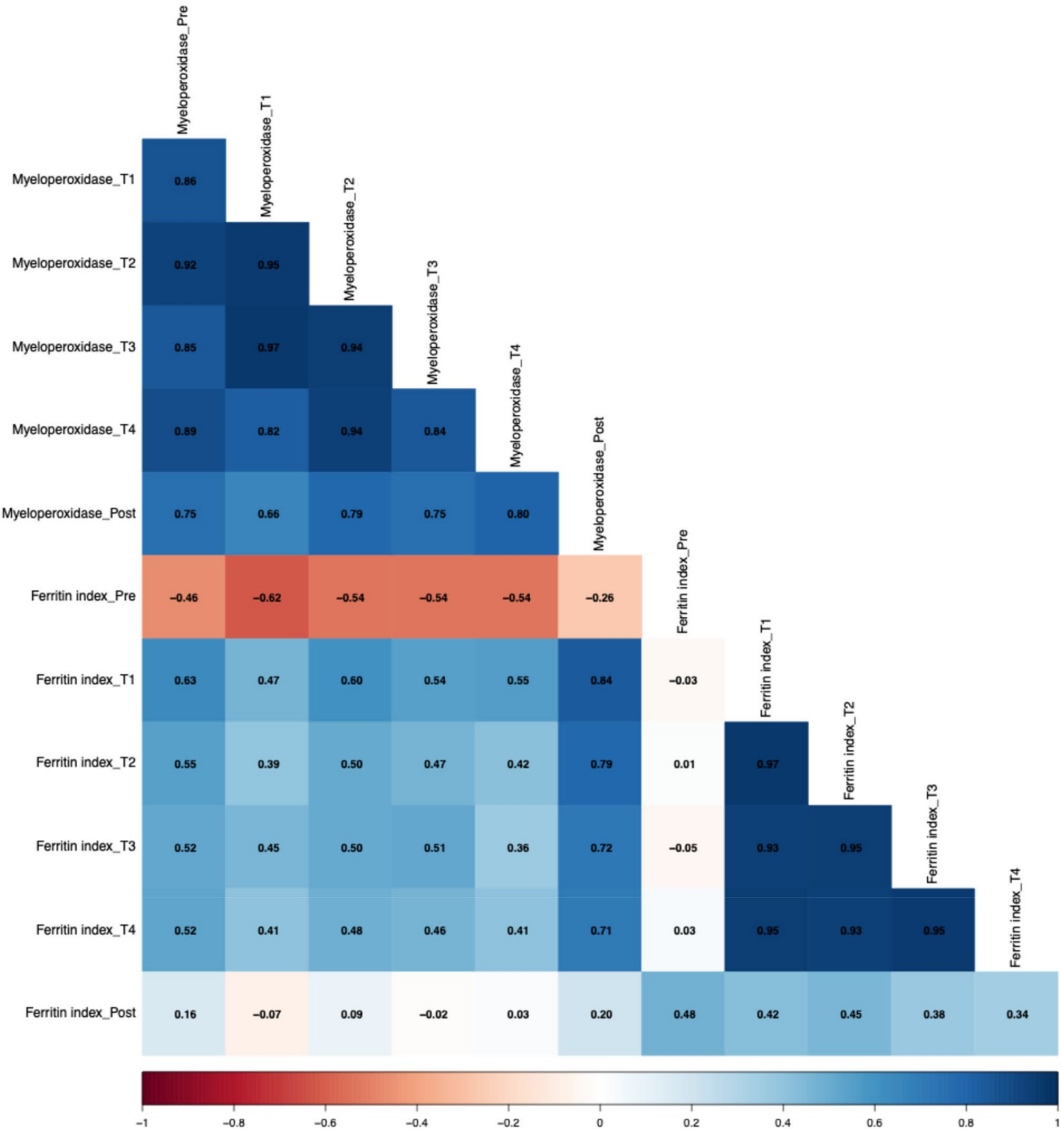
Spearman correlations between the ferritin index and myeloperoxidase.

Spearman correlations between MPO and ferritin index are demonstrated in Fig. 5. Negative associations were found between the ferritin index at pre-measurement and MPO at different time points pre (r = − 0.46, *p* > 0.05), T1 (r = − 0.62, *p* < 0.05), T2 (r = − 0.54, *p* < 0.05), T3 (r = − 0.54, *p* < 0.05) and T4 (r = 0.54, *p* < 0.05). Positive correlations were found between MPO at post-measurement and the ferritin index at T1 (r = 0.84, *p* < 0.001), T2 (r = 0.79, *p* < 0.001), T3 (r = 0.72, *p* < 0.01) and T4 (r = 0.71, *p* < 0.01).

**Fig. 6:**
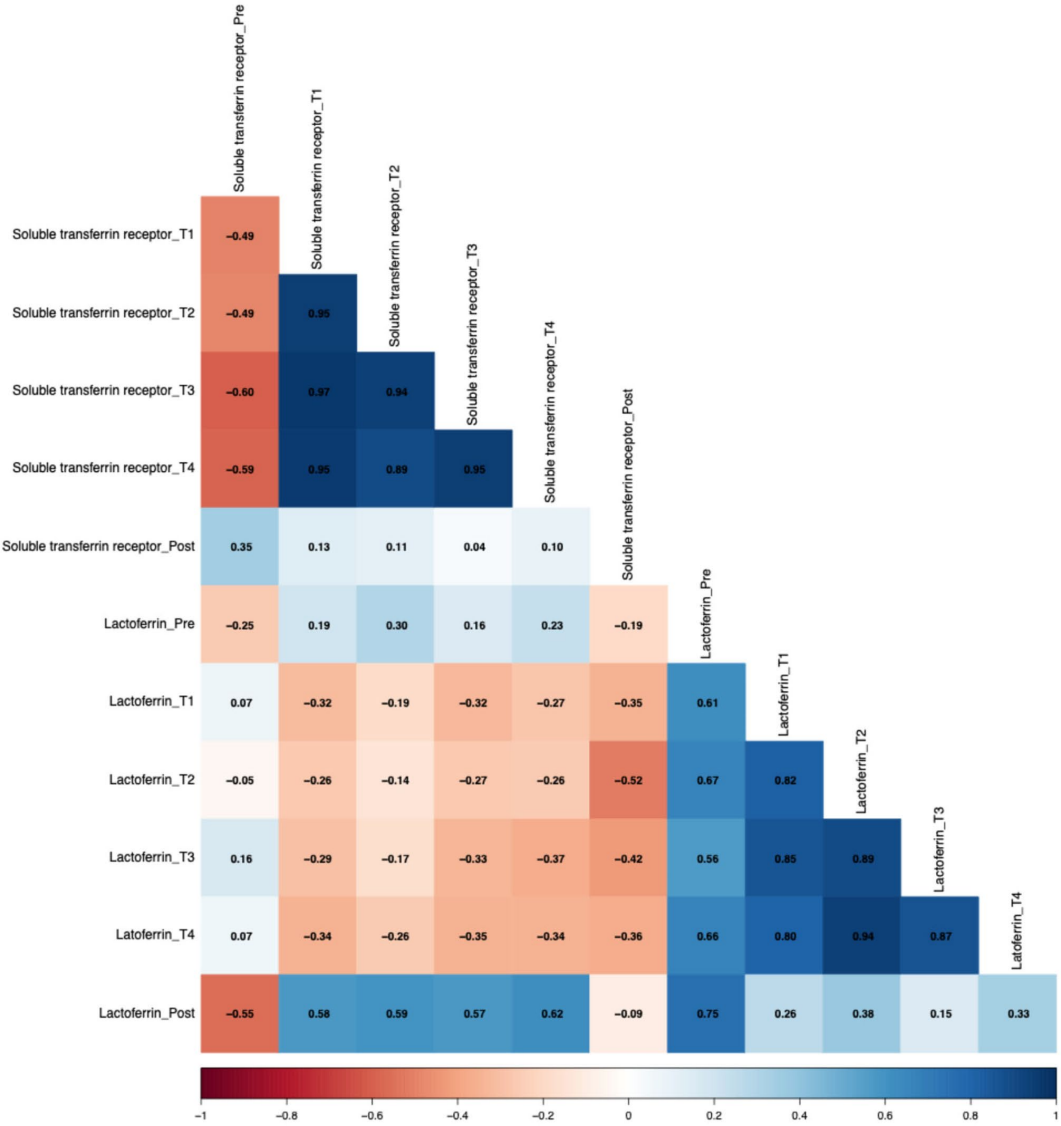
Spearman correlations between soluble transferrin receptor and lactoferrin.

The relationship between sTfR and lactoferrin differed across the various measurement points (Fig. 6). At altitude, positive correlations were found between sTfR levels at T1 (r = 0.58, *p* < 0.05), T2 (r = 0.59, *p* < 0.05), T3 (r = 0.57, *p* < 0.05), and T4 (r = 0.62, *p* < 0.05) with lactoferrin at the post-measurement. In contrast, a negative correlation was observed between pre-measurement sTfR and lactoferrin levels at the post-measurement (r = − 0.55, *p* < 0.05).

**Fig. 7:**
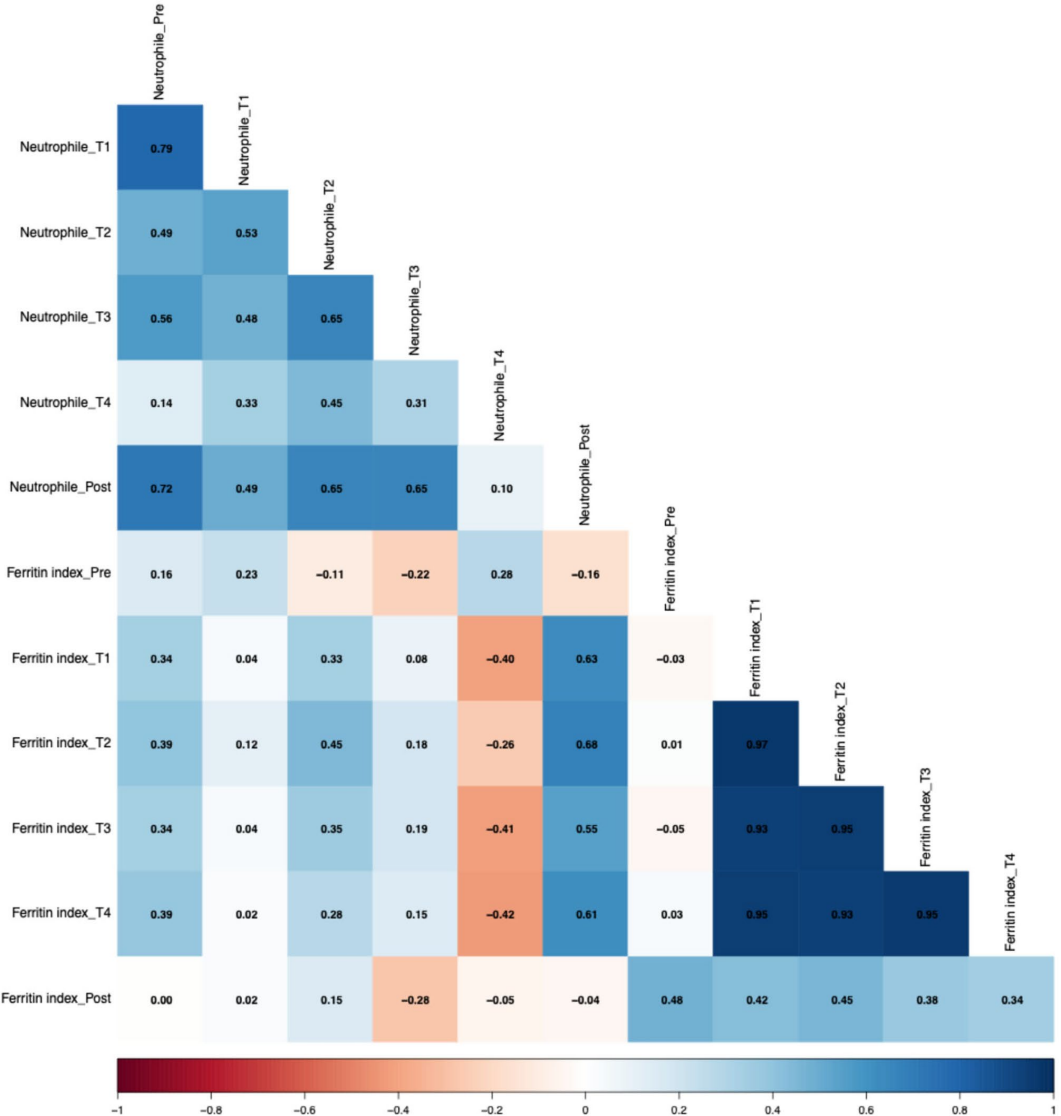
Spearman correlations between the number of neutrophils and ferritin index.

Associations between the number of neutrophils and the ferritin index are shown in Fig. 7. Negative correlations were observed between the number of neutrophils at T4 and the ferritin index at different time points, with r = − 0.52 for T1, r = − 0.26 for T2, r = − 0.42 for T3, and r = − 0.42 for T4 (*p* > 0.05). Conversely, the post-measurement neutrophil count showed positive correlations with the ferritin index at these same time points, with T1 (r = 0.63, *p* < 0.05), T2 (r = 0.68, *p* < 0.01), T3 (r = 0.55, *p* < 0.05), and T4 (r = 0.61, *p* < 0.05).

### Granger causalities between hematological, iron metabolism and immune function variables

**Fig. 8:**
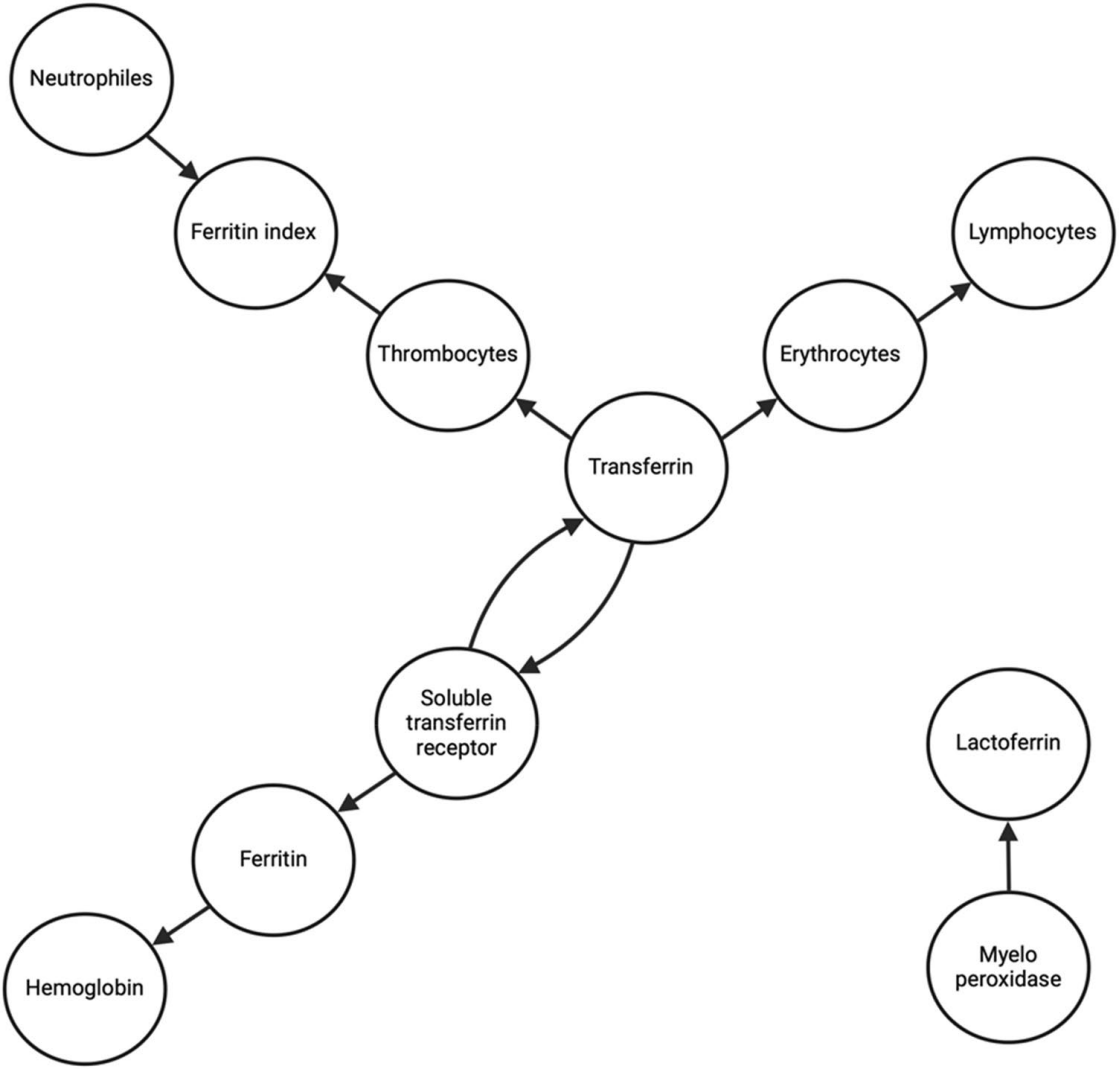
Granger causality network of homological, iron metabolism and immunological variables.

We applied the Granger causality test to see whether prior values of the measured parameters could predict future values of those. The analysis revealed significant unidirectional or bidirectional relationships between various hematological, immune, and iron metabolism parameters, as visualized in the network diagram (Fig. 8). Each arrow represents a Granger-causal connection, highlighting how these biological systems might interact. (Fig. 8). All reported influences are statistically significant.

In the Granger causality network analysis, erythrocytes emerged as a significant predictor for lymphocytes (F = 16.25, *p* < 0.01), indicating an influence of erythrocytes on lymphocyte dynamics. Transferrin exerted a predictive effect on both thrombocytes (F = 26.91, *p* < 0.001) and erythrocytes (F = 698.44, *p* < 0.001). Ferritin was found to predict hemoglobin concentration (F = 25.44, *p* < 0.001), while ferritin itself was influenced by sTfR (F = 14.14, *p* < 0.05). Moreover, sTfR and transferrin demonstrated a bidirectional relationship (sTfR—> transferrin: F = 13.81, *p* < 0.05; transferrin—> sTfR: F = 15.68, *p* < 0.05), highlighting a mutual predictive influence between these two parameters. Neutrophils (F = 46.06, *p* < 0.001) and thrombocytes (F = 21.58, *p* < 0.001) both showed predictive effects on the ferritin index, indicating their involvement in ferritin index modulation. Additionally, myeloperoxidase (MPO) predicted lactoferrin (F = 76.05, *p* < 0.001), though notably, both MPO and lactoferrin are functionally isolated from the main network, suggesting a distinct subnetwork or independent regulatory pathway.

## Discussion

After an average of 21 days of normobaric hypoxia, VO_2_max increased significantly. However, large interindividual differences were observed and no correlation of VO_2_max increases with training load.

Hematological variables showed marked adjustments, including increases in platelets, reticulocytes, and hemoglobin concentration during altitude, while erythropoietin only increased in the first 24 h. Regarding iron metabolism, ferritin concentration increased initially but dropped again by the end of the measurements, while sTfR showed a significant increase. MPO and lactoferrin decreased but showed correlations with iron metabolism variables. Finally, network and Granger causality analyses revealed bidirectional interactions between central hematological and metabolic parameters, with MPO and lactoferrin showing functional isolation within a subnetwork.

The performance of the athletes, assessed through VO_2_max, increased following the training camp in normobaric hypoxia, though there was high interindividual variability. This variability has been widely documented in the literature and appears to depend on genetics, baseline fitness levels, nutritional status, and individual acclimatization capacity^2,14^. The extent to which the VO_2_max changed is similar to that observed in comparable studies in hypobaric hypoxia suggesting that both, training camps in normobaric and hypobaric hypoxia, may elicit comparable adaptation effects on endurance performance^15,16^.

Hematological adaptations were also consistent with findings from studies in hypobaric hypoxia, showing an increase in hemoglobin concentration and erythrocytes^17^. The increase of sTfR and ferritin index ties erythropoiesis to adaptive responses in iron metabolism. Increased iron demand is met through suppression of hepcidin—the central regulator of systemic iron homeostasis^18,19^—mobilization of stored iron, and upregulation of intestinal iron transport. These mechanisms ensure the body has sufficient resources to sustain the hematological adaptations necessary for improved oxygen transport capacity. These findings are supported by detailed molecular and systemic studies, reinforcing the centrality of iron metabolism in hypoxic adaptation^7,20,21^. However, it must be taken into account that iron intake was not controlled in this study and that inter-individual differences may also be affected by this.

A systemic physiological connection between hematological changes and the immune system is indicated by the negative correlation between the ferritin index at pre-measurement and MPO over time. These associations might reflect the iron dependency on neutrophil function, indicated by MPO^22^. However, positive correlations were found between the ferritin index and MPO at all time points at altitude. These findings suggest that an increased iron demand at earlier time points may be linked to a subsequent rise in inflammatory activity, potentially as an adaptive response to altered iron availability^23^. To further understand how iron-related immune responses are modulated under hypoxic conditions lactoferrin was included in the analysis. As a neutrophil-derived glycoprotein with antimicrobial and iron-scavenging properties, lactoferrin offers insights into neutrophil-mediated iron handling. In parallel, sTfR correlates with the receptors expressed on cells that are “fishing” for iron. The expression of this receptor is upregulated when there is a higher iron demand in these cells^24^. Associations between these markers disclose an interesting interplay. Based on our data, two patterns were identified. The first is that lactoferrin at pre- and post-measurements show positive correlations with sTfr at altitude (T1–T4), with lactoferrin at post displaying moderately significant associations. The second pattern demonstrated that lactoferrin at altitude (T1–T4) shows only small but constant negative correlation with sTfr at altitude. The first pattern indicated that higher sTfR levels during altitude exposure correlated with an increase in lactoferrin levels at the end of the exposure period, reflecting adaptive changes in iron metabolism in response to altitude. This assumption is underlined through the fact that, even though correlations are not significant, there is also a negative correlation between pre-lactoferrin and sTfr at altitude. The second pattern suggests a shift in the relationship between these markers in normobaric hypoxia exposure. The interplay between those markers seems to be the same at sea level, only changing under normobaric hypoxia. The hypoxic conditions might reduce lactoferrin as a marker of neutrophil activity as a response to an increased cellular demand for iron. The positive correlations between the number of neutrophils at post-measurement and the ferritin index at altitude (T1–T4) might reflect that an increased number of neutrophils results in an increase of the ferritin index. Neutrophils also express transferrin receptors (TfRs) to ensure iron uptake for their activity in pathogen defense^22^. The increased sTfR values, consequently, result in an elevated ferritin index.

Some of the correlations can be explained more precisely by the Granger causality analysis, which provides a statistical framework that discloses potential directional influences between the parameters we measured. Granger analysis reveals a structure centered around transferrin, with three main branches extending from it and an additional subnetwork involving lactoferrin and myeloperoxidase. The central position of transferrin underscores the importance of iron availability in the body’s adaptation to normobaric hypoxia, as transferrin is recognized as a key regulator for iron homeostasis^25^.

The upper left branch illustrates the interplay between the innate immune response and iron metabolism (Fig. 8). Here, neutrophils and thrombocytes are shown to predict the ferritin index, suggesting the iron demands of both these cell types. This increased demand may drive the elevated expression of transferrin receptors, particularly on neutrophils, where they facilitate iron uptake and support neutrophil functions such as ROS production during pathogen defense^22^. Furthermore, thrombocytes are known to express transferrin receptor 2 (TfR2), which binds diferric transferrin and might influence platelet activation, count, and size^26^. Specifically, the inverse correlation between transferrin saturation and thrombocyte function may reflect a regulatory mechanism, as higher transferrin saturation has been associated with reduced platelet aggregation and activation. These findings highlight a coordinated role for transferrin in balancing iron metabolism across immune and hemostatic systems, linking iron availability with cellular function and homeostasis.

The lower left branch represents the hematological system’s dependency on iron (Fig. 8). The bidirectional relationship between transferrin and soluble sTfR suggests a feedback mechanism where increased iron demand, reflected in elevated sTfR levels, stimulates transferrin production to facilitate iron transport^27,28^. sTfR predicts ferritin levels, as higher receptor expression indicates enhanced iron uptake at the cellular level, mobilizing ferritin-bound iron stores^29^. This mobilization suggests the prioritization of erythropoiesis during iron deficiency or physiological demands, such as altitude adaptation, where ferritin predicts hemoglobin concentration by providing the iron reserves required for hemoglobin synthesis^30^. These findings emphasize the intricate regulatory mechanisms that govern iron metabolism, ensuring adequate erythropoiesis under varying physiological conditions.

The right branch highlights transferrin’s critical role in predicting erythrocyte levels, emphasizing iron availability as a cornerstone of red blood cell production. Transferrin acts as the primary plasma iron transporter, delivering iron to erythroid precursors via TfRs, which are crucial for hemoglobin synthesis and erythropoiesis^31^. This relationship demonstrates a shared dependency of erythroid cells and lymphocytes on iron, as both require sufficient iron availability for optimal proliferation and function. At altitude, the prioritization of erythropoiesis to meet oxygen transport demands could explain the downstream effects on lymphocyte populations. This interplay is potentially mediated by EPO signaling, which activates both erythrocytes and lymphocytes through the EPO receptor (EPO-R). The response of these two very different cell types to the same growth signal could be explained by the need to maintain a relatively constant ratio even under extreme conditions, such as hypoxic stress. Erythroid precursors may exhibit a higher sensitivity for EPO, enabling them to respond more rapidly and efficiently to hypoxic stress, which aligns with the prioritization of erythropoiesis during hypoxia^32^. This dual influence of EPO on erythroid and lymphoid cells underscores the interconnected nature of the hematological and immune systems, especially under conditions of increased physiological demand, such as high altitude. Lastly, the subnetwork involving lactoferrin and MPO, with MPO predicting lactoferrin, aligns well with their respective roles in neutrophil granule function. MPO, as a component of the primary (azurophilic) granules, is among the first antimicrobial agents released during neutrophil activation and is critical for pathogen destruction but can also cause significant collateral damage and release free iron into the environment^33,34^. In contrast, lactoferrin, stored in the secondary granules, is released subsequently. This process mitigates the damage caused by MPO through its iron-binding capability, which sequesters free iron and contributes to microbial growth inhibition by depriving pathogens of this essential resource^35,36^. Thus, lactoferrin not only complements MPO’s antimicrobial effects but also helps maintain immune balance by reducing potential tissue damage through iron binding.

All data and results are derived from experiments conducted under normobaric hypoxia. Therefore, any interpretation or translation to hypobaric hypoxia should be approached with caution. Due to the stringent scheduling to the training and competition planning of elite athletes, it was necessary to adhere to their individualized training programs rather than implementing a standardized training protocol during a camp in normobaric hypoxia. This may have introduced variability in training loads and adaptations across participants. Furthermore, logistical limitations restricted recruitment to three distinct groups of athletes, resulting in a relatively small sample size of 15 individuals, which may limit the generalizability of our findings. Additionally, total hemoglobin mass (tHb) was not measured due to practical and logistical constraints, representing a further limitation of this study. This may imply that hemoglobin concentration from our data could be influenced through plasma reduction which occurs in hypoxic conditions^37^. We acknowledge that hemoglobin concentration and changes in iron metabolism are also affected by other factors than normobaric hypoxia, such as iron status before the camp. To analyze this a control group would have been necessary, which we were not able to provide due to limited opportunities for intervention within the rigid seasonal planning of the athletes. We recognize the need for caution when interpreting the network due to several limitations. Granger causality cannot always distinguish direct from indirect effects and relies on temporal predictability rather than mechanistic proof. Additionally, the limited number of time points restricts the analysis to time lags of 1. Despite these limitations, it serves as an initial tool for gaining deeper and novel insights into the underlying mechanisms and for generating hypotheses for future studies.

## Conclusion

The study highlights the importance of considering additional physiological systems, especially iron metabolism and the immune system, for a more comprehensive understanding of adaptation to normobaric hypoxia. The strong dependence of erythropoiesis on iron availability presents a significant challenge for maintaining adaptive immune function, which is crucial for athletes’ health and performance. In practical sports applications, this highlights the importance of monitoring iron metabolism and immune function alongside hematology, training, and performance assessments. Additionally, ensuring continuous and sustainable iron availability for athletes is essential to support performance adaptations, particularly in endurance athletes using (normobaric) hypoxia to enhance performance. Future research should explore larger cohorts and incorporate measurements of total hemoglobin mass to better understand interindividual variability in adaptations. Moreover, mechanistic studies linking iron metabolism, immune function, and performance outcomes could refine altitude training protocols, contributing to more personalized approaches in sports science.

## Data Availability

All data is available upon request. Please reach out to Svenja.Nolte@sport.uni-giessen.de.
